# Outcomes of endoscopic balloon dilatation for *de novo* and anastomotic Crohn’s strictures: a Canadian tertiary center experience

**DOI:** 10.1093/ecco-jcc/jjag172

**Published:** 2026-11-04

**Authors:** Sudheer Kumar Vuyyuru, Gaurav Nigam, Shane W Goodwin, Shivya Sethi, Jamie Gregor, Brian Yan, Michael Sey, Vipul Jairath

**Affiliations:** Department of Medicine, Division of Gastroenterology, Western University, London, ON, Canada; Translational Gastroenterology and Liver Unit, Nuffield Department of Medicine, John Radcliffe Hospital, University of Oxford, Oxford, United Kingdom; Lawson Health Research Institute, Western University, London, ON, Canada; Lawson Health Research Institute, Western University, London, ON, Canada; Department of Medicine, Division of Gastroenterology, Western University, London, ON, Canada; Department of Medicine, Division of Gastroenterology, Western University, London, ON, Canada; Department of Medicine, Division of Gastroenterology, Western University, London, ON, Canada; Lawson Health Research Institute, Western University, London, ON, Canada; Division of Epidemiology and Biostatistics, Western University, London, ON, Canada; Department of Medicine, Division of Gastroenterology, Western University, London, ON, Canada; Lawson Health Research Institute, Western University, London, ON, Canada; Division of Epidemiology and Biostatistics, Western University, London, ON, Canada

**Keywords:** stenosis, obstruction, dilatation, Crohn’s disease, fibrosis

## Abstract

**Background:**

Over half of patients with Crohn’s disease (CD) develop intestinal strictures that often require surgical resection. Endoscopic balloon dilatation (EBD) can be a potential alternative therapeutic option.

**Methods:**

This retrospective cohort study included adult patients with CD (≥18 years) who underwent EBD between January 2016 and September 2024 at two Canadian hospitals. The primary outcome was technical success. Secondary outcomes were clinical success at 6 and 12 months, hospitalization due to obstruction, and CD-related surgery. Cox proportional hazards models assessed predictors of outcomes.

**Results:**

A total of 234 patients (age: 47.6 years, males: 47.4%) were included with mean disease duration of 17.7 years and median follow-up of 61 months. A majority of the strictures (226/234) were dilated under ileocolonoscopy guidance, and 79.4% had symptoms at baseline. Most strictures were of *de novo* type (62.5%) and the median number of stricture dilatations was 3 (IQR: 2). Technical success was achieved in 81% (150/185). Clinical success was noted in 47.7% at 6 months. The cumulative probabilities of being hospitalization-free were 96.9% (95% CI 94.7-99.2]) at 1 year, 91.3% (95% CI 87.5-95.2) at 3 years, and 85.0% (95% CI 79.8-90.5) at 5 years. The cumulative probabilities of being surgery-free were 91.1% (95% CI 87.5-94.9) at 1 year, 81.7% (95% CI 76.5-87.2) at 3 years, and 78.1% (95% CI 72.4-84.3) at 5 years. Multiple strictures were associated with an increased risk of surgery while anastomotic strictures and maximum balloon diameter were associated with lower risk. No patients experienced procedure-related complications.

**Conclusions:**

EBD is effective and safe in *de novo* and anastomotic strictures with the majority of patients achieving technical success and being free of surgery within 5 years.

WHAT YOU NEED TO KNOW **Background** 

- Endoscopic balloon dilatation (EBD) of CD-related strictures may serve as a potential alternative to surgery in carefully selected patients
- However, high-quality evidence supporting its use remains limited

**Findings** 

- EBD is effective and safe for both *de novo* and anastomotic CD-related strictures

**Implications for patient care** 

- Our study adds valuable evidence on both short- and long-term outcomes of EBD for CD-related strictures in the post-biologic era

## 1. Introduction

Crohn’s disease (CD) is a chronic inflammatory bowel disease (IBD) that can affect any segment of the gastrointestinal tract. It is characterized by periods of remission and relapse, with patients often developing strictures as a result of prolonged transmural inflammation leading to fibrosis.^1^^,^^2^ Recent data from a large cohort study showed that approximately one-third of patients with CD present with a stricturing phenotype.^3^ The development of strictures in CD is a common and progressive complication, with approximately 13%-20% of CD patients developing small bowel strictures within 5 years of diagnosis.^4^^,^^5^ It is estimated that almost half of patients with CD will develop strictures in their lifetime.^6^ Patients with stricturing CD experience greater disease burden, increased use of immunosuppressants, higher rates of CD-related symptoms, extraintestinal manifestations, and surgeries compared to those with an inflammatory phenotype.^3^ This in turn contributes to substantial economic burden on healthcare systems.^7^

Untreated CD-related strictures can result in symptoms such as abdominal pain and bloating and often cause intestinal obstruction which may sometimes need emergency bowel resection. The management of strictures secondary to CD includes medical therapy, endoscopic interventions, and surgery.^6^ The choice of intervention depends on stricture characteristics such as stricture type (inflammatory or fibrotic), location, length, and severity of bowel narrowing. Surgical stricture resection is highly effective for CD-related strictures but is not a definitive therapy and the risk of recurrence of stricture at the anastomotic site remains. Endoscopic balloon dilatation (EBD) has emerged as a minimally invasive, bowel-preserving option for treating strictures. Studies have shown that EBD is effective and well-tolerated, reducing the need for surgical intervention. Evidence also suggests that EBD is a cost-effective strategy for managing CD strictures.^8^ Despite being minimally invasive, patient-preferred, and simple to perform, there are uncertainties related to technique, patient selection, frequency of follow-up procedures, and long-term efficacy.

This lack of evidence underscores the need for further investigation into the long-term efficacy and safety of EBD, as there is no clear consensus on the optimal management strategy for CD strictures. This study aims to provide comprehensive insights into the real-world effectiveness of EBD in a Canadian population by analyzing its efficacy as well as safety.

## 2. Methods

### 2.1. Study population and design

This is a retrospective cohort study of patients with a confirmed and established diagnosis of CD based on standard clinical, endoscopic, and histologic criteria. Eligible participants were adult patients (≥18 years) with CD who underwent EBD through either ileocolonoscopy or small bowel enteroscopy between January 2016 and September 2024, at London Health Sciences Center and St. Joseph’s Health Center in London, Canada. Patients with strictures due to conditions other than CD, dilatation for ileal pouch strictures, stomal strictures and insufficient procedure details were excluded. The reporting of this study follows the STROBE (Strengthening the Reporting of Observational Studies in Epidemiology) statement.^9^

### 2.2. Protocol for endoscopic balloon dilatation

The balloon dilatations were performed by five gastroenterologists specialized in managing IBD, each with more than 10 years of experience in diagnostic and therapeutic endoscopic procedures. The decision to proceed with EBD was made by the treating gastroenterologist. Strictures shorter than 5 cm were considered suitable for balloon dilatation. Computed tomography (CT) or magnetic resonance (MR) enterography was the standard cross-sectional imaging used to characterize strictures prior to EBD in most patients. There was no fixed protocol dictating the interval between imaging and endoscopic treatment; imaging was often deferred in cases of short, fibrotic strictures where the endoscopist could confidently characterize the stricture directly at ileocolonoscopy. Antegrade dilatation was performed for most strictures using a wire-guided controlled radial expansion (CRE) balloon catheter, with graded dilatation starting at the lowest balloon diameter and progressing serially to the desired target diameter.

### 2.3. Definitions and outcomes

The primary outcome was technical success, which was defined as the ability to pass the endoscope through the stricture after dilatation without immediate complications. Secondary outcomes were clinical success, defined as absence of symptoms and no need for additional interventions (surgery or repeat dilation) at 6 and 12 months post-procedure, complication rates such as perforation, bleeding, or infection as documented in patient records, time to hospitalization due to intestinal obstruction, and CD-related surgery because of stricture. Data on baseline characteristics, procedural details, and follow-up outcomes were extracted from electronic patient records.

### 2.4. Ethical considerations

Ethical approval for medical chart review was granted by the Western University Research Ethics Board through a delegated and full board review, providing waiver of need for patient consent.

### 2.5. Statistical analysis

Potential risk factors associated with risk of CD-related surgery (bowel resection because of CD-related indications) and CD-related hospitalization were evaluated, and cumulative probabilities for both outcomes were calculated at years 1, 3, and 5. Cox proportional hazards models examined differences in the time to long-term CD-related outcomes across selected clinical risk factors. To report differences in relative risk of maintenance or improvement of symptoms at 6 and 12 months we utilized modified Poisson regression.^10^ The factor of interest was whether dilatation was a technical success. As a sensitivity analysis, an alternative definition of technical success was included using a maxium balloon diameter of >13.5 mm. All models were adjusted for potential confounders using baseline characteristics (sex, smoking status, age, biologic use, baseline symptomatic, pre-dilatation scope passage, maximum balloon dilatation size, presence of multiple strictures, type of strictures, and presence of strictures during endoscopy). Characteristics found to be significant at an alpha level of 0.20 in univariate models were kept in the final multivariable model, following literature recommendations and supplemented with review by clinical expertise to ensure important characteristics were not missed. Due to the high correlation between the definition of technical success and maximum balloon dilatation, a sensitivity analysis was conducted excluding maximum balloon size from the model. All analyses were conducted using R v.4.4.1.^11^

## 3. Results

### 3.1. Baseline characteristics

A total of 281 patients were screened and 234 were included in the final analysis after elimination of patients who met the exclusion criteria (baseline characteristics are presented in Table 1). Among the included patients 46.4% (*n* = 111) were males and mean disease duration was 17.7 (SD ± 12.3) years. The majority had an ileocolonic disease location (59.8%) followed by ileal (35.1%) and colonic (5.1%) disease location. Strictures were *de novo* (primary) in the majority of cases (62.8%, *n* = 147) and anastomotic type in 37.5% (*n* = 71). The median number of stricture dilatations was 3 (IQR: 2). The most common location of *de novo* strictures was the terminal ileum (28.5%, *n* = 66) followed by ileocecal valve (18.5%, *n* = 43). Over half of the patients included (66.2%, *n* = 155) were on advanced therapies at baseline and the majority of patients were symptomatic at baseline (79.4%). The median time from dilatation to follow-up was 61 months (interquartile range [IQR] = 57).

**Table 1.** Baseline characteristics of patients with stricturing Crohn’s disease.

Characteristic	%/Mean (SD)/median (IQR)
**Age (years)**	47.6 (15.2)
**Sex (male)**	47.4% (111)
**Duration (years)**	17.7 (12.3)
**Disease location**	
** Ileal**	35.0% (88)
** Ileocolonic**	59.8% (140)
** Colonic**	5.1% (12)
** Upper GI involvement**	6.0% (14)
**History of intestinal fistula**	16.7% (39)
**Stricture type (*de novo*)**	62.8% (147)
**Stricture location**	
** Anastomosis**	37.5% (87)
** IC valve**	18.5% (43)
** Colonic**	8.2% (19)
** TI**	28.5% (66)
** Ileum**	5.6% (14)
** Rectal**	1.7% (4)
**Smoking status**	
** Never**	48.9% (113)
** Current**	20.4% (47)
** Former**	20.4% (47)
** Unknown**	10.4% (24)
**Multiple strictures**	16.2% (38)
**Advanced therapies at baseline**	66.2% (155)
**Type of baseline therapy**	
** Anti-IL12/23**	17.1%
** Anti-integrin**	9.4%
** Anti-TNF**	35.5%
** IL-23**	4.3%
** None**	33.8%
**Previous bowel resection**	53.9% (125)
**Symptoms at baseline**	79.4% (185)

Abbreviations: GI: gastrointestinal, IC valve: ileocolonic valve, IL: interleukin, TI: terminal ileum, TNF: tumor necrosis factor.

### 3.2. Procedure-related outcomes

In the majority of patients, the strictures were accessible to ileocolonoscopy, with eight patients undergoing double balloon enteroscopy for balloon dilatation. Only one-third of strictures (32.5%) were considered purely fibrotic on visual inspection without overt features of inflammation, with the remaining strictures having at least one ulcer at the site of stricture. The endoscope was able to traverse the stricture before dilatation in only 9.4% of cases. The median balloon dilatation of the stricture was 15 mm (IQR = 1.5). In 31 patients, two strictures were noted during the endoscopy procedure and in seven patients three strictures were noted. Technical success was considered only if all strictures were successfully dilated with subsequent ability to pass the scope beyond the most distal stricture. The overall technical success rate was 81% (150/185). Using an alternative definition based on balloon diameter (≥13.5 mm) the technical success rate was similar (78.2% [183/234]). Technical success rate in patients with two or more strictures was 42% (16/38). None of the patients experienced complications such as perforation or bleeding (Table 2).

**Table 2.** Stricture-related characteristics and procedure outcomes.

Procedure outcomes	*n*, %, SD, IQR
**Median number of dilatations during follow-up (IQR)**	3 (2)
**Endoscopic appearance of strictures**	
** Fibrotic**	32.5% (76)
** Strictures with ulcers**	58.1% (136)
** Not reported**	9.4% (22)
**Ability to pass score across stricture pre-dilatation**	
** Yes**	9.4% (22)
** No**	79.9% (187)
** Not reported**	10.7% (25)
**Median maximum balloon diameter (mm) (IQR)**	15 (1.5)
**Technical success^a^**	81% (150)
**Technical success (≥13.5 mm)**	
** Yes**	78.2%
** No**	21.8%
**Complications**	0%

a Data available for 185 patients.

Abbreviation: IQR: interquartile range.

### 3.3. Short-term symptomatic improvement

At baseline, 79.4% (185/233) had symptoms of abdominal pain and/or dietary avoidance. At 6 months, 47.7% (83/174) achieved clinical success, whereas 46.8% (83/177) of patients achieved clinical success at 12 months. On modified Poisson regression, no major predictors were significant in predicting symptomatic improvement at 6 or 12 months except for the presence of baseline symptoms (for 6 month outcome) (Table 3). Similar results were observed by using an alternative definition of technical success based on balloon diameter (Table S1). No differences in estimates were found when omitting maximum balloon dilatation size from models.

**Table 3.** Factors predicting symptomatic improvement at 6 or 12 months.

Characteristic	6-month improvement (*n* = 123)		12-month improvement (*n* = 128)	
	RR (95% CI)	*P*-value	RR (95% CI)	*P*-value
**Technical success (ref = no)**	1.07 (0.59-1.96)	.81	1.13 (0.66-1.93)	.66
**Maximum balloon diameter (mm)**	0.99 (0.85-1.14)	.87	1.03 (0.91-1.16)	.66
**Age**	1.01 (0.99-1.02)	.36	0.99 (0.98-1.01)	.28
**Sex (ref = Male)**	1.39 (0.86-2.26)	.18	1.17 (0.74-1.86)	.49
**Stricture type (ref = *de novo*)**	1.19 (0.74-1.91)	.48	1.14 (0.71-1.84)	.58
**Smoking status (ref = never)**				
** Current**	0.79 (0.41-1.51)	.48	0.82 (0.51-1.33)	.42
** Former**	0.60 (0.30-1.18)	.14	0.58 (0.30-1.11)	.10
**Multiple strictures (ref = no)**	0.99 (0.53-1.88)	.98	1.23 (0.78-1.92)	.37
**Baseline advanced therapy (ref = no)**	1.12 (0.62-2.01)	.70	1.13 (0.68-1.88)	.64
**Baseline symptoms (ref = no)**	0.52 (0.35-0.80)	.002	0.77 (0.50-1.19)	.24
**Stricture type on endoscopy (ref = fibrotic)**	0.98 (0.61-1.57)	.93	0.99 (0.63-1.56)	.97
**Ability to pass scope before dilatation (ref = no)**	0.49 (0.15-1.56)	.23	1.57 (0.90-2.75)	.11

### 3.4. Long-term risk of surgery

The cumulative probabilities of being surgery-free was 91.1% (95% confidence interval [CI]: 87.5%-94.9%) at 1 year, 81.7% (95% CI: 76.5%-87.2%) at 3 years, and 78.1% (95% CI: 72.4%-84.3%) at 5 years (see Figure 1 and Table 4). Individuals with multiple strictures were significantly more likely to require CD-related surgery compared to those without multiple strictures (hazard ratio [HR] 2.45, 95% CI [1.13-5.31], *P* = .02) (Table 5). No differences in results were obtained when using an alternative definition of technical success requiring a dilatation of at least 13.5 mm (Table S2). In the full model, achievement of technical success neared statistical significance (HR: 0.31, 95% CI: 0.09-1.01, *P* = .05), which became statistically significant after exclusion of maximum balloon dilatation from the model (HR: 0.38, 95% CI: 0.18-0.79, *P* = .01; Table S3).

**Figure 1.**
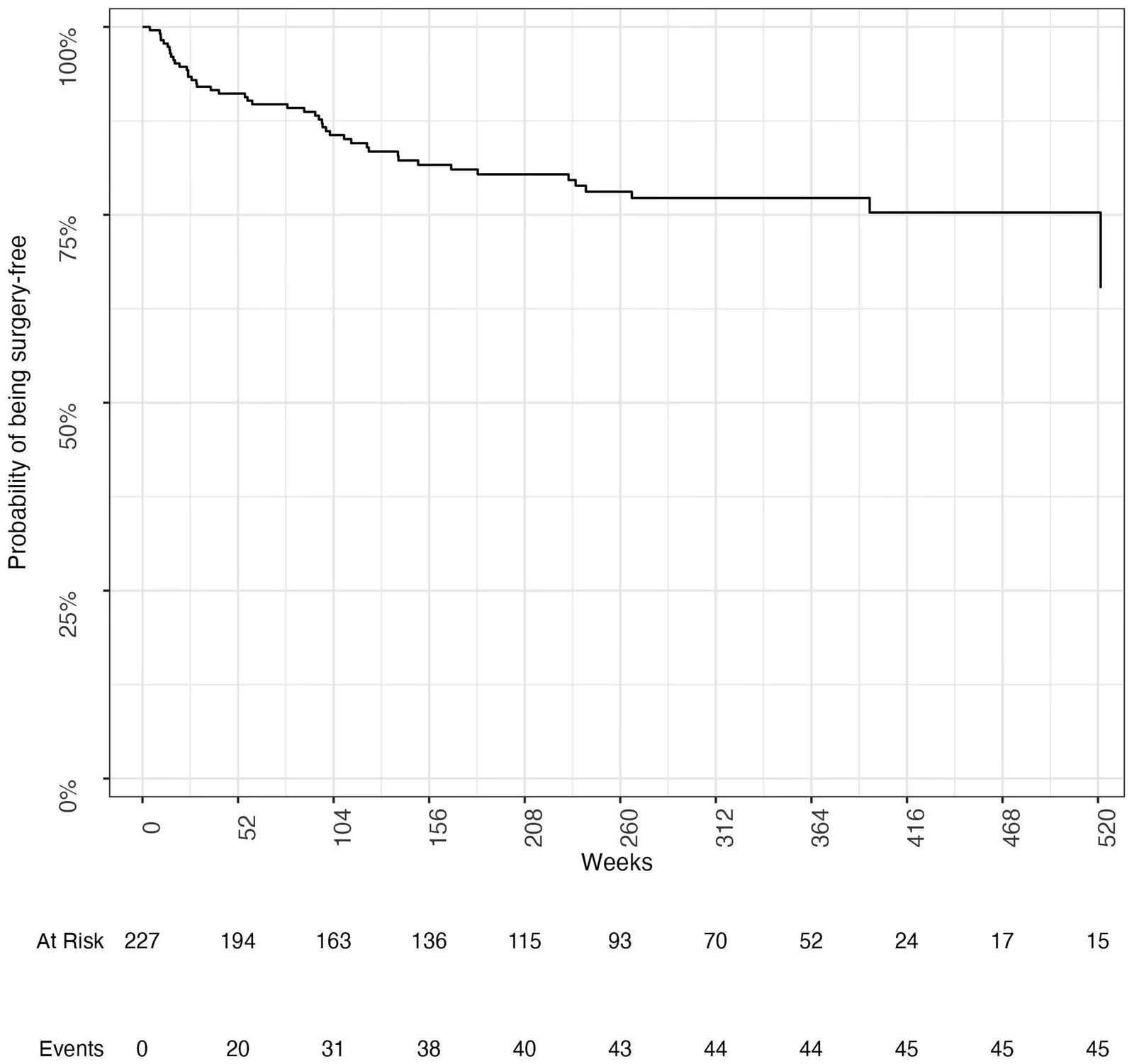
Kaplan–Meier plot showing the probability of being free of CD-related surgeries.

**Table 4.** Long-term outcomes following endoscopic balloon dilatation.

Key outcomes	*n*, %
**Hospitalization**	14.1%
**Surgery**	20.3%
**Clinical success^a^ at 6 months**	
** Yes**	35.6%
** No**	39.1%
** Unknown**	25.3%
**Clinical success^a^ at 12 months**	
** Yes**	35.5%
** No**	40.2%
** Unknown**	24.4%
**Median time from dilatation to last follow-up (months)**	61 (57)
**Median time from dilatation to hospitalization (months)**	25 (42)
**Median time from dilatation to surgery (months)**	18 (25)

a Clinical success: absence of symptoms and no need for additional interventions (surgery or repeat dilatation).

**Table 5.** Factors predicting hospitalization due to intestinal obstruction and surgery.

Characteristic	Risk of hospitalization (*n* = 134)		Risk of surgery (*n* = 133)	
	HR (95% CI)	*P*-value	HR (95% CI)	*P*-value
**Technical success (ref = no)**	0.41 (0.16-1.06)	.07	0.52 (0.20-1.34)	.18
**Maximum dilatation (mm)**	1.09 (0.92-1.29)	.33	**0.80 (0.64-1.00)**	**.049**
**Age**	**1.03 (1.00-1.06)**	**.04**	0.99 (0.96-1.02)	.67
**Sex (ref = M)**	1.74 (0.72-4.17)	.22	1.19 (0.52-2.74)	.68
**Stricture type (ref = *de novo*)**	1.14 (0.46-2.83)	.77	**0.32 (0.12-0.91)**	**.03**
**Smoking (ref = never)**				
** Current**	1.10 (0.35-3.40)	.87	2.10 (0.74-6.00)	.16
** Former**	1.83 (0.70-4.78)	.22	2.26 (0.67-7.57)	.19
**Multiple strictures (ref = no)**	0.92 (0.32-2.63)	.88	**3.44 (1.48, 8.03)**	**.004**
**Baseline advanced therapy (ref = no)**	**0.35 (0.16-0.81)**	**.01**	1.00 (0.40-2.51)	.99
**Baseline symptomatic (ref = no)**	2.34 (0.63-8.66)	.20	1.08 (0.34-3.47)	.90
**Stricture type on endoscopy (ref = fibrotic)**	1.22 (0.45-3.31)	.70	1.88 (0.75-4.74)	.18
**Ability to pass scope before dilatation (ref = no)**	0.26 (0.45-3.31)	.22	1.02 (0.22-4.67)	.98

Values in bold indicate statistical significance.

### 3.5. Long-term risk of hospitalization due to intestinal obstruction

The cumulative probabilities of being hospitalization-free were 96.9% (95% CI: 94.7%-99.2%) at 1 year, 91.3% (95% CI: 87.5%-95.2%) at 3 years, and 85.0% (95% CI: 79.8%-90.5%) at 5 years (see Figure 2). Full details on the risk of hospitalization can be found in Table 2. Older individuals were significantly more likely to require hospitalization compared to younger individuals (HR: 1.03, 95% CI: 1.00-1.06, *P* = .02) (Table 5). Additionally, those on an advanced therapy at baseline were significantly less likely to require hospitalization compared to those off advanced therapies (HR: 0.45, 95% CI: 0.20-0.98, *P* = .04). No differences in results were obtained when using an alternative definition of technical success requiring a dilatation of at least 13.5 mm (Table S2) or after exclusion of maximum balloon dilataion from the model (Table S3).

**Figure 2.**
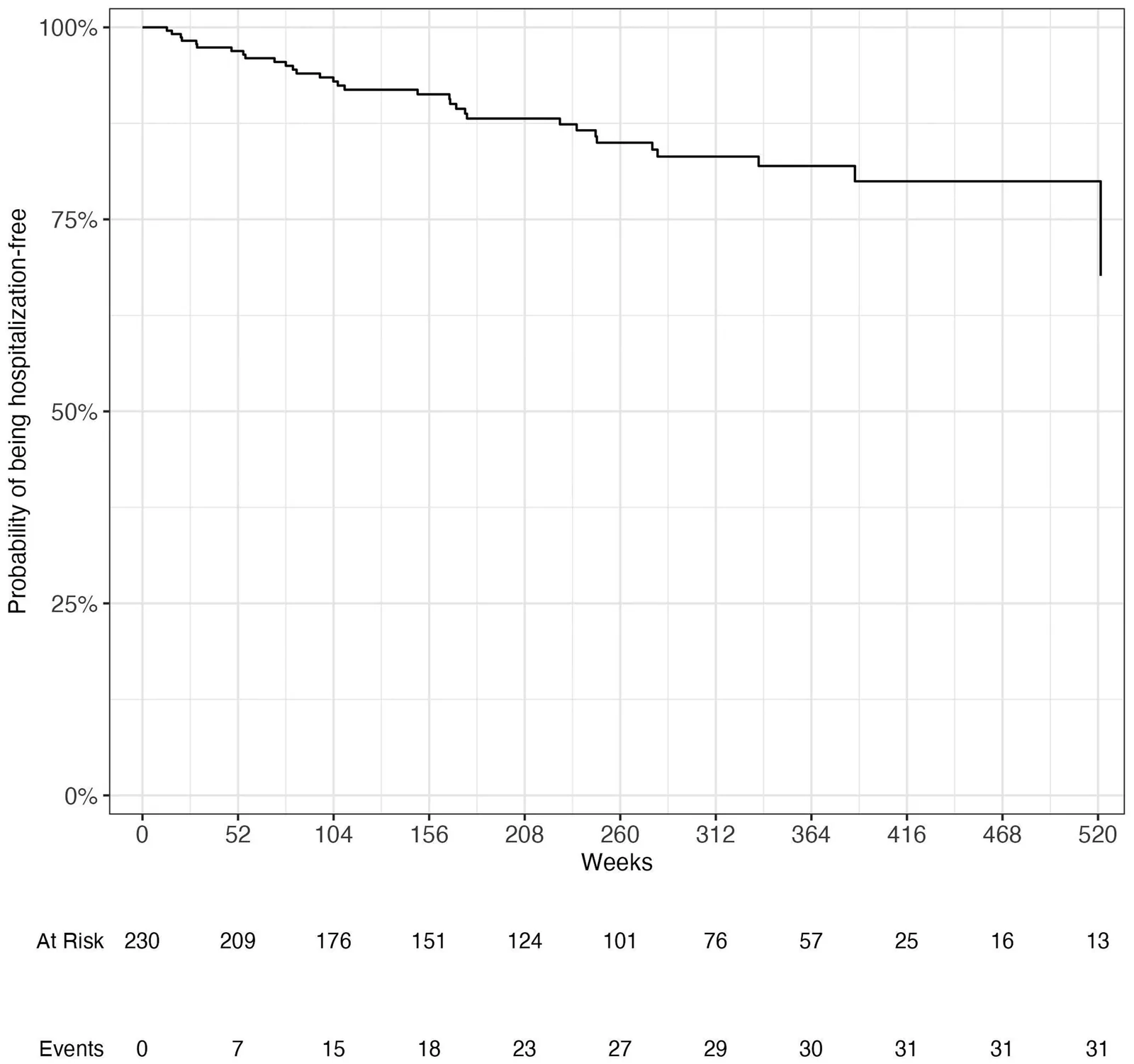
Kaplan–Meier plot showing the probability of being free of hospitalization due to obstruction.

### 3.6. Factors influencing outcomes

We assessed the interaction between technical success and various characteristics of strictures (inflammatory vs fibrotic stricture, anastomotic vs *de novo*), and presence of symptoms at baseline, on long term risk of surgery and hospitalization. We observed an effect of technical success of EBD on time to surgery, but time to hospitalization was not significantly modified by nature of stricture, type of strictures, or presence of symptoms at baseline (Tables S4, S5, and S6). We also evaluated the risk of repeat EBD within 6 months of the index procedure. The presence of symptoms at baseline was associated with an increased risk of repeat EBD within 6 months, whereas the presence of multiple strictures was associated with a lower risk (Table S7). However, as the decision to perform repeat dilatation was at the discretion of the treating physician, these findings should be interpreted with caution.

## 4. Discussion

Our study represents one of the largest observational cohorts evaluating both short- and long-term outcomes following EBD in patients with CD-related intestinal strictures. With a median follow-up of 61 months (IQR 57), our cohort provides one of the longest reported observation periods in the EBD literature to date, allowing for more robust characterization of 3- and 5-year outcomes than has previously been possible in most published series. The key findings demonstrate that EBD is a safe and effective intervention, with a high rate of technical success and favorable clinical outcomes, in both *de novo* and anastomotic strictures. Among the identified predictors, the presence of multiple strictures was associated with an increased risk of subsequent surgery, whereas anastomotic strictures were associated with a lower surgical risk compared with *de novo* strictures following EBD. In addition, a larger diameter of balloon was independently associated with a reduced risk of surgery, suggesting a potential impact of procedural factors on long-term outcomes.

Since initial reports of EBD usage in CD in the 1980s,^12^ EBD has emerged as a potential alternative interventional therapeutic option to surgery for the management of CD-related intestinal strictures. However, the available evidence supporting its effectiveness is largely derived from observational studies.^13^ Procedural characteristics such as balloon diameter, duration of inflation, and endoscopist experience vary widely across centers, and there remains no uniform agreement regarding the optimal EBD technique. Similarly, type of outcomes evaluated across studies varies significantly given a lack of unform standardization. Overall EBD is generally considered effective, with technical success rates ranging from 62% to 100% in previously published studies.^14^ Technical success is the commonly assessed procedure-related outcome measure and is generally defined as the ability to traverse the stricture with an adult or pediatric colonoscope following balloon dilatation. In our study, owing to the retrospective nature, technical success was not explicitly documented in approximately one-fifth of patients. To address this limitation, we adopted an alternative and objective definition of technical success, achievement of a balloon diameter of at least 13.5 mm, exceeding the outer diameter of an adult colonoscope. Using this revised definition, the overall technical success rate in our cohort was 78%, which is consistent with previously reported data.^14^ From a safety standpoint, balloon dilatation is generally regarded as a safe procedure, with an pooled major complication rate of 4% and iatrogenic perforation rate of 3%.^14^ In our study notably we did not observe any procedure-related complications, such as bleeding or perforation. This favorable safety profile can be partly explained by the relatively lower median maximum balloon diameter (15 mm) used in our cohort compared with studies reporting higher complication rates (18-25 mm).^14^

There is no clear consensus on the ideal balloon diameter that should be targeted while doing EBD. Expert consensus from the Global Interventional Inflammatory Bowel Disease Group recommends dilatation to 18-20 mm and smaller balloon if stricture diameter is <1 cm,^15^ whereas a more recent RAND-based expert consensus suggests a target diameter of 15-18 mm.^16^ Moreover, there is conflicting evidence on the relationship between maximum balloon diameter and clinical benefit. In a retrospective study by Reutemann et al. ,failure to achieve a maximum dilatation of 14 mm or more increased the risk of surgery (HR 2.88, 95% CI [1.10-7.53]).^17^ While there was no difference in the risk of future surgery between maximum EBD sizes of 14-15 and 16-18 mm, those reaching 16-18 mm exhibited a longer interval between subsequent dilatations. On the contrary, in a pooled analysis, balloon diameter did not influence the risk of surgery.^18^ Although, in our study maximum balloon diameter predicted lower risk of surgery, it should be noted that mean diameter in our study is lower compared to previously published studies.

Common symptoms encountered by patients with CD-related strictures are abdominal pain, cramping, nausea, vomiting, and bloating, with acute intestinal obstructive symptoms occurring in more severe cases. Dietary avoidance is another important and common symptom often neglected by physicians. Importantly, these symptoms are subjective and do not always correlate with the degree of luminal narrowing. In a recently reported cohort, there were no statistically significant differences in bloating (53.6% vs 61.5%, *P* = .69), post-prandial pain (57.6% vs 25.0%, *P* = .14), or vomiting (10.0% vs 20.0%, *P* = .47) between patients with definitive versus patients without strictures although these analyses were limited by small sample sizes.^19^ This can be explained by an influence of dietary patterns on symptom burden. Moreover, there is currently no standardized, validated symptom assessment tool that exists that is specifically designed for this population. In the STRIDENT trial,^20^ the first randomized controlled trial evaluating advanced therapies in stricturing CD, an obstructive symptom (OS) score based on a five-point Likert pain scale over the preceding 14 days was developed. Similarly, the CREOLE study introduced the Crohn’s Disease Obstructive Score (CDOS).^21^ However, these scores have not been validated. Therefore, there is a need for a dedicated, validated symptom assessment tool in patients with CD-related strictures. In our study, we assessed symptoms such as abdominal pain, dietary avoidance, and observed favorable short-term clinical outcomes, with symptomatic improvement at 6 months in 47% of patients. Lower numbers may be because we only considered improvement if it was reported that the patients were symptom free.

Despite the short-term effectiveness of EBD, a significant proportion of patients with CD-related strictures ultimately require surgical intervention. Previous studies have demonstrated that EBD can delay the need for surgery by 6.45 years.^22^ In our cohort, the cumulative rate free of surgery at 5 years was 78.1%, which aligns with previously published literature. A multicenter study from Italy involving 46 patients reported a technical success rate of 100% and demonstrated that surgery was avoided in 92.6% of patients over a mean follow-up of 40.7 months.^23^ Similarly, a study from Japan reported cumulative surgery-free rates of 79% at 2 years and 73% at 3 years, with significantly higher surgery-free survival among patients with successful EBD compared to those with unsuccessful procedures.^24^ These findings are further supported by meta-analytic data. Navaneethan et al., in a meta-analysis of 24 studies comprising 1163 patients undergoing EBD, reported a surgical intervention rate of 27% over a median follow-up of 15-70 months.^14^

Several factors have been associated with an increased risk of surgery following EBD, including stricture length, type of stricture, and location of stricture. Importantly, stricture length ≤5 cm was associated with a significantly lower risk of surgery, and every 1-cm increase of stricture length is associated with an increased hazard for surgery by 8%.^18^ Another important factor that has been shown to impact long-term outcomes in CD-related surgery was use of biologic therapies. A systematic review and meta-analysis by Nascimento Filho et al. indicated that the use of biologics and achieving endoscopic remission prior to EBD were linked to a lower risk of subsequent surgery.^25^ Strictures in CD are generally considered as a combination of fibrotic with underlying chronic inflammation, which is an ongoing driver of extracellular matrix deposition and recurrence of strictures. Therefore, effective control of inflammation through biological therapies is crucial. However, studies evaluating the efficacy of these therapies in the stricturing phenotype are limited. In a prospective single arm study evaluating the effectiveness of adalimumab in symptomatic stricturing CD (CREOLE),^21^ two-thirds of patients achieved treatment success defined as adalimumab continuation without prohibited treatment (corticosteroids after the 8 weeks following inclusion, other anti-tumor necrosis factor agents), endoscopic dilatation, or bowel resection at week 24. A recent randomized controlled trial (STRIDENT)^20^ comparing the efficacy of intensive (adalimumab + thiopurine) vs standard therapy (adalimumab alone) in patients with symptomatic CD-related strictures also demonstrated less treatment failure and greater improvement in stricture MaRIA scores, indicating that CD strictures are responsive to advanced therapies. Similarly, post-hoc analyses of newer advanced therapies such as upadacitinib and risankizumab showed improvement in intestinal stenosis.^26^

We acknowledge several limitations inherent to our study. First, the retrospective design introduces the potential for selection bias. In addition, technical success of EBD was not consistently documented for subsequent dilatation procedures. Although our multivariable models were adjusted for baseline biologic exposure, changes in medical therapy following EBD including initiation, escalation, or switching of biologic or advanced therapies were made at the discretion of the treating physicians and were not systematically captured or incorporated into outcome analyses. Notably, in our cohort, 66% of patients were receiving advanced therapies at baseline, which increased to 88.8% at follow-up. While this variability may confound the observed associations, it also reflects real-world clinical practice in the contemporary biologic era. Furthermore, the proportion of patients with small bowel strictures in our cohort was relatively low, which limits the generalizability of our findings to this subgroup. Detailed and standardized measurements of stricture length were not available; however, at our center, balloon dilatation is typically attempted only for short-segment strictures measuring ≤5 cm. Accordingly, the strictures included in this study are presumed to be short in length, consistent with current clinical practice and guideline recommendations. Symptom assessment was based on medical records, so it is possible that some patients classified as asymptomatic might have had mild, unreported symptoms. Although subgroup analyses did not demonstrate a significant interaction between technical success and stricture-related factors, including stricture characteristics, stricture type, and the presence of symptoms, the study may have been underpowered to reliably detect such interaction. Moreover, in our study cross-sectional imaging parameters were not systematically captured, precluding their incorporation into a validated composite phenotypic classification. Standardized cross-sectional imaging protocols should be further evaluated in future prospective studies of EBD. Despite these limitations, our study provides important and clinically relevant insights into the short- and long-term outcomes of EBD for CD-related intestinal strictures in a real-world setting. The large sample size, extended follow-up over 5 years, and inclusion of patients managed in the post-biologic era strengthen the relevance of our findings and support the role of EBD as a safe and effective therapeutic option in appropriately selected patients.

## Supplementary Material

jjag172_Supplementary_Data

## Data Availability

Data, analytic methods, and study materials will be made available to other researchers on reasonable request to the corresponding author.

## References

[1] Lin X , Wang Y, Liu Z, et al Intestinal strictures in Crohn’s disease: a 2021 update. Ther Adv Gastroenterol. 2022;15:17562848221104951. 10.1177/17562848221104951 PMC9218441 35757383

[2] Cosnes J , Gower-Rousseau C, Seksik P, Cortot A. Epidemiology and natural history of inflammatory bowel diseases. Gastroenterology. 2011;140:1785-1794. 10.1053/j.gastro.2011.01.055 21530745

[3] Fan Y , Zhang L, Omidakhsh N, et al Patients with stricturing or penetrating Crohn’s disease phenotypes report high disease burden and treatment needs. Inflamm Bowel Dis. 2023;29:914-922. 10.1093/ibd/izac162 PMC10233399 35880838

[4] Rieder F , Zimmermann EM, Remzi FH, Sandborn WJ. Crohn’s disease complicated by strictures: a systematic review. Gut. 2013;62:1072-1084. 10.1136/gutjnl-2012-304353 PMC4884453 23626373

[5] Burisch J , Kiudelis G, Kupcinskas L, et al; Epi-IBD group. Natural disease course of Crohn’s disease during the first 5 years after diagnosis in a European population-based inception cohort: an Epi-IBD study. Gut. 2019;68:423-433. 10.1136/gutjnl-2017-315568 29363534

[6] Rieder F , Fiocchi C, Rogler G. Mechanisms, management, and treatment of fibrosis in patients with inflammatory bowel diseases. Gastroenterology. 2017;152:340-350.e6. 10.1053/j.gastro.2016.09.047 PMC5209279 27720839

[7] Fan Y , Zhang L, Jiménez MC, et al Economic burden related to fistulas or strictures among commercially insured patients with Crohn’s disease in the United States. J Manag Care Spec Pharm. 2023;29:400-408. 10.18553/jmcp.2023.29.4.400 PMC10387957 36989446

[8] Lee KE , Lim F, Faye AS, Shen B, Hur C. Endoscopic balloon dilation is cost-effective for Crohn’s disease strictures. Dig Dis Sci. 2022;67:5462-5471. 10.1007/s10620-022-07420-z 35290570

[9] Vandenbroucke JP , von Elm E, Altman DG, et al; STROBE Initiative. Strengthening the reporting of observational studies in epidemiology (STROBE): explanation and elaboration. PLoS Med. 2007;4:e297. 10.1371/journal.pmed.0040297 PMC2020496 17941715

[10] Zou GY , Donner A. Extension of the modified Poisson regression model to prospective studies with correlated binary data. Stat Methods Med Res. 2013;22:661-670. 10.1177/0962280211427759 22072596

[11] R: The R Project for Statistical Computing. Accessed January 26, 2026. https://www.r-project.org/

[12] Brower RA. Hydrostatic balloon dilation of a terminal ileal stricture secondary to Crohn’s disease. Gastrointest Endosc. 1986;32:38-40. 10.1016/S0016-5107(86)71728-8 2868966

[13] Morar PS , Faiz O, Warusavitarne J, et al; Crohn’s Stricture Study (CroSS) Group. Systematic review with meta-analysis: endoscopic balloon dilatation for Crohn’s disease strictures. Aliment Pharmacol Ther. 2015;42:1137-1148. 10.1111/apt.13388 26358739

[14] Navaneethan U , Lourdusamy V, Njei B, Shen B. Endoscopic balloon dilation in the management of strictures in Crohn’s disease: a systematic review and meta-analysis of non-randomized trials. Surg Endosc. 2016;30:5434-5443. 10.1007/s00464-016-4902-1 27126619

[15] Shen B , Kochhar G, Navaneethan U, et al Practical guidelines on endoscopic treatment for Crohn’s disease strictures: a consensus statement from the Global Interventional Inflammatory Bowel Disease Group. Lancet Gastroenterol Hepatol. 2020;5:393-405. 10.1016/S2468-1253(19)30366-8 31954438

[16] Bettenworth D , Baker ME, Fletcher JG, et al; Stenosis Therapy Anti-Fibrotic Research (STAR) Consortium. A global consensus on the definitions, diagnosis and management of fibrostenosing small bowel Crohn’s disease in clinical practice. Nat Rev Gastroenterol Hepatol. 2024;21:572-584. 10.1038/s41575-024-00935-y PMC12063211 38831007

[17] Reutemann BA , Turkeltaub JA, Al-Hawary M, Waljee AK, Higgins PDR, Stidham RW. Endoscopic balloon dilation size and avoidance of surgery in stricturing Crohn’s disease. Inflamm Bowel Dis. 2017;23:1803-1809. 10.1097/MIB.0000000000001181 PMC5599343 28691940

[18] Bettenworth D , Gustavsson A, Atreja A, et al A pooled analysis of efficacy, safety, and long-term outcome of endoscopic balloon dilation therapy for patients with stricturing Crohn’s disease. Inflamm Bowel Dis. 2017;23:133-142. 10.1097/MIB.0000000000000988 28002130

[19] Saunders B , Lesack K, Rosentreter R, et al Comparing obstructive symptoms and dietary restrictions with Crohn’s disease stricture severity. J Crohns Colitis. 2024;18:i1429. 10.1093/ecco-jcc/jjad212.0901

[20] Schulberg JD , Wright EK, Holt BA, et al Intensive drug therapy versus standard drug therapy for symptomatic intestinal Crohn’s disease strictures (STRIDENT): an open-label, single-centre, randomised controlled trial. Lancet Gastroenterol Hepatol. 2022;7:318-331. 10.1016/S2468-1253(21)00393-9 34890567

[21] Bouhnik Y , Carbonnel F, Laharie D, et al; GETAID CREOLE Study Group. Efficacy of adalimumab in patients with Crohn’s disease and symptomatic small bowel stricture: a multicentre, prospective, observational cohort (CREOLE) study. Gut. 2018;67:53-60. 10.1136/gutjnl-2016-312581 PMC5754855 28119352

[22] Lian L , Stocchi L, Remzi FH, Shen B. Comparison of endoscopic dilation vs surgery for anastomotic stricture in patients with Crohn’s disease following ileocolonic resection. Clin Gastroenterol Hepatol. 2017;15:1226-1231. 10.1016/j.cgh.2016.10.030 27816758

[23] de’Angelis N , Carra MC, Borrelli O, et al Short- and long-term efficacy of endoscopic balloon dilation in Crohn’s disease strictures. WJG. 2013;19:2660-2667. 10.3748/wjg.v19.i17.2660 PMC3645384 23674873

[24] Hirai F , Beppu T, Takatsu N, et al Long-term outcome of endoscopic balloon dilation for small bowel strictures in patients with Crohn’s disease. Dig Endosc. 2014;26:545-551. 10.1111/den.12236 24528293

[25] Menezes Nascimento Filho H , Kum AST, Bestetti AM, et al Patient-related factors associated with long-term outcomes after successful endoscopic balloon dilation for Crohn’s disease-associated Ileo-colic strictures: a systematic review and meta-analysis. Crohns Colitis 360. 2024;6:otae041. 10.1093/crocol/otae041 PMC11339545 39175792

[26] Lu C , Feagan BG, Fletcher JG, et al; Stenosis Therapy and Anti-Fibrotic Research Consortium. Management of small bowel Crohn’s disease strictures: to cut, to stretch, or to treat inflammation? Gastroenterology. 2024;167:1283-1291.e1. 10.1053/j.gastro.2024.08.030 39222715

