# Supplementary material for: Outcomes of endoscopic balloon dilatation for de novo and anastomotic Crohn’s strictures: a Canadian tertiary center experience
Source: J Crohns Colitis. 2026 Nov 4;20(10):jjag172. doi: 10.1093/ecco-jcc/jjag172 (PMC13735507; doi:10.1093/ecco-jcc/jjag172)
Supplement: Supplementary file 1 [file jjag172_supplementary_data.zip › Supplementary_tables.docx]

**Supplementary Table 1.** Relative Risk for the symptomatic improvement compared to baseline using alternate definition of technical success.

| **Characteristic** | **6-month outcome (n=123)** |  | **12-month outcome (n=128)** |  |
| --- | --- | --- | --- | --- |
|  | **RR (95% CI)** | **p-value** | **RR (95% CI)** | **p-value** |
| Technical Success (ref = no) | 0.67 (0.33-1.38) | 0.28 | 1.26 (0.58-2.71) | 0.56 |
| Max Dilation (mm) | 1.04 (0.88-1.22) | 0.66 | 1.00 (0.86-1.17) | 0.96 |
| Age | 1.01 (0.99-1.02) | 0.26 | 0.99 (0.98-1.01) | 0.39 |
| Sex (ref = male) | 1.39 (0.90-2.16) | 0.14 | 1.26 (0.81-1.95) | 0.30 |
| Stricture Type (ref = denovo) | 1.27 (0.81-1.99) | 0.29 | 1.00 (0.63-1.60) | 0.99 |
| Smoke (ref = never)  Current  Former | 0.73 (0.40-1.34)  0.67 (0.37-1.21) | 0.31  0.18 | 0.84 (0.53-1.32)  0.62 (0.33-1.17) | 0.45  0.14 |
| Multiple Strictures (ref = no) | 1.04 (0.58-1.89) | 0.89 | 1.30 (0.82-2.05) | 0.27 |
| Baseline Advanced Therapy (ref = no) | 1.04 (0.61-1.76) | 0.89 | 1.30 (0.80-2.12) | 0.30 |
| Baseline Symptoms (ref = no) | 0.52 (0.35-0.78) | 0.001 | 0.70 (0.46-1.06) | 0.09 |
| Stricture Type on Endoscopy (ref = fibrotic) | 0.85 (0.55-1.32) | 0.48 | 0.95 (0.61-1.47) | 0.80 |
| Ability to Pass Scope Before Dilatation (ref = no) | 0.36 (0.11-1.17) | 0.09 | 1.23 (0.66-2.31) | 0.51 |

**Supplementary Table 2.** Risk of CD-related surgery and hospitalization in patients with Crohn’s disease using alternate definition of technical success.

| **Characteristic** | **CD-related Surgery (n=162)** |  | **Hospitalization (n=162)** |  |
| --- | --- | --- | --- | --- |
|  | **HR (95% CI)** | **p-value** | **HR (95% CI)** | **p-value** |
| Technical Success (ref = no) | 0.31 (0.09-1.01) | 0.053 | 1.30 (0.30-5.56) | 0.72 |
| Max Dilation (mm) | 1.05 (0.82-1.36) | 0.68 | 1.07 (0.85-1.34) | 0.59 |
| Age | 0.99 (0.96-1.01) | 0.29 | **1.03 (1.00-1.06)** | **0.02** |
| Sex (ref = male) | 1.51 (0.73-3.09) | 0.26 | 1.68 (0.76-3.74) | 0.20 |
| Stricture Type (ref = denovo) | 0.61 (0.27-1.37) | 0.23 | 1.02 (0.43-2.40) | 0.96 |
| Smoke (ref = never)  Current  Former | 1.58 (0.68-3.72)  1.89 (0.71-4.99) | 0.29  0.20 | 1.78 (0.67-4.75)  1.94 (0.75-5.04) | 0.25  0.17 |
| Multiple Strictures (ref = no) | **2.45 (1.13-5.31)** | **0.02** | 1.01 (0.36-2.84) | 0.98 |
| Baseline Advanced Therapy (ref = No) | 1.05 (0.49-2.26) | 0.89 | **0.45 (0.20-0.98)** | **0.04** |
| Baseline Symptoms (ref = no) | 1.53 (0.51-4.60) | 0.45 | 2.17 (0.61-7.66) | 0.21 |
| Stricture Type on Endoscopy(ref = fibrotic) | 1.37 (0.62-3.02) | 0.43 | 1.37 (0.52-3.58) | 0.52 |
| Ability to Pass Scope Before Dilatation (ref = no) | 0.59 (0.13-2.58) | 0.48 | 0.25 (0.03-2.14) | 0.21 |

**Supplementary Table 3.** Risk of CD-related surgery and hospitalization in patients with Crohn’s disease, excluding maximum balloon dilation size from models.

| **Characteristic** | **Hospitalization (n=162)** |  | **CD-related Surgery (n=162)** |  |
| --- | --- | --- | --- | --- |
|  | **HR (95% CI)** | **p-value** | **HR (95% CI)** | **p-value** |
| Technical Success (ref = No) | 1.69 (0.56-5.09) | 0.35 | **0.38 (0.18-0.79)** | **0.01** |
| Age | **1.03 (1.00-1.06)** | **0.02** | 0.99 (0.96-1.01) | 0.32 |
| Sex (ref = M) | 1.77 (0.82-3.84) | 0.15 | 1.55 (0.76-3.15) | 0.23 |
| Stricture Type (ref = Denovo) | 1.00 (0.43-2.35) | 1.00 | 0.60 (0.27-1.34) | 0.22 |
| Smoke (ref = Never)  Current  Former | 1.78 (0.67-4.71)  1.90 (0.73-4.89) | 0.25  0.19 | 1.58 (0.68-3.70)  1.79 (0.71-4.53) | 0.29  0.22 |
| Multiple Strictures (ref = No) | 1.00 (0.36-2.79) | 1.00 | **2.48 (1.15-5.35)** | **0.02** |
| Baseline Advanced Therapy (ref = No) | **0.44 (0.20-0.97)** | **0.04** | 1.04 (0.49-2.22) | 0.92 |
| Baseline Symptomatic (ref = No) | 2.20 (0.63-7.72) | 0.22 | 1.56 (0.52-4.68) | 0.43 |
| Stricture Type on Endoscopy (ref = Fibrotic) | 1.40 (0.54-3.65) | 0.49 | 1.40 (0.64-3.06) | 0.40 |
| Ability to Pass Scope Before Dilatation (ref = No) | 0.27 (0.03-2.23) | 0.23 | 0.60 (0.14-2.60) | 0.49 |

**Supplementary Table 4:** Risk of CD-related surgery and hospitalization in patients with Crohn’s disease including an interaction between Technical success and Inflammatory vs Fibrotic strictures.

| **Characteristic** | **CD-related Surgery** |  | **Hospitalization** |  |
| --- | --- | --- | --- | --- |
|  | **HR (95% CI)** | **p-value** | **HR (95% CI)** | **P-value** |
| Technical Success (ref = No) | 0.45 (0.08—2.42) | 0.35 | 0.84 (0.08—8.49) | 0.89 |
| Max Dilation (mm) | 1.05 (0.81—1.36) | 0.70 | 1.06 (0.85—1.33) | 0.61 |
| Age | 0.99 (0.96-1.01) | 0.25 | **1.03 (1.00-1.06)** | **0.02** |
| Sex (ref = M) | 1.50 (0.73—3.08) | 0.27 | 1.71 (0.77—3.80) | 0.19 |
| Stricture Type (ref = Denovo) | 0.62 (0.27—1.39) | 0.24 | 1.02 (0.43—2.41) | 0.96 |
| Smoke (ref = Never) |  |  |  |  |
| Current | 1.59 (0.68—3.73) | 0.29 | 1.76 (0.66—4.69) | 0.26 |
| Former | 1.92 (0.73—5.07) | 0.19 | 1.94 (0.75—5.06) | 0.17 |
| Multiple Strictures (ref = No) | **2.37 (1.09—5.15)** | **0.03** | 1.02 (0.36—2.86) | 0.97 |
| Baseline Advanced Therapy (ref = No) | 1.02 (0.47—2.21) | 0.96 | **0.45 (0.20—0.98** | **0.045** |
| Baseline Symptomatic (ref = No) | 1.51 (0.50—4.55) | 0.47 | 2.15 (0.61—7.60) | 0.24 |
| Stricture Type on Endoscopy (ref = Fibrotic) | 1.96 (0.48—7.90) | 0.35 | 0.83 (0.08—8.78) | 0.88 |
| Ability to Pass Scope Before Dilatation (ref = No) | 0.57 (0.13—2.53) | 0.46 | 0.25 (0.03—2.16) | 0.21 |
| Technical Success x Stricture Type of Stricture on Endoscopy (ref = Fibrotic) | 0.60 (0.12—3.05) | 0.54 | 1.77 (0.15—21.3) | 0.65 |

**Supplementary Table 5**: Risk of CD-related surgery and hospitalization in patients with Crohn’s disease including an interaction between Technical success and De-novo vs Anastomatic strictures.

| **Characteristic** | **CD-related Surger** |  | **Hospitalization** |  |
| --- | --- | --- | --- | --- |
|  | **HR (95% CI)** | **p-value** | **HR (95% CI)** | **P-value** |
| Technical Success (ref = No) | 0.33 (0.09—1.15) | 0.08 | 0.45 (0.09—2.14) | 0.31 |
| Max Dilation (mm) | 1.06 (0.82—1.36) | 0.67 | 1.05 (0.83—1.32) | 0.69 |
| Age | 0.99 (0.96-1.01) | 0.29 | **1.03 (1.00-1.06)** | **0.02** |
| Sex (ref = M) | 1.52 (0.74—3.13) | 0.26 | 1.65 (0.75—3.62) | 0.22 |
| Stricture Type (ref = Denovo) | 0.69 (0.19—2.47) | 0.57 | 0.13 (0.01—1.34) | 0.09 |
| Smoke (ref = Never) |  |  |  |  |
| Current | 1.60 (0.68—3.77) | 0.28 | 1.94 (0.72—5.23) | 0.19 |
| Former | 1.92 (0.72—5.14) | 0.19 | 2.26 (0.86—5.97) | 0.10 |
| Multiple Strictures (ref = No) | **2.44 (1.13—5.29)** | **0.02** | 0.97 (0.34—2.74) | 0.96 |
| Baseline Advanced Therapy (ref = No) | 1.06 (0.49—2.29) | 0.87 | **0.42 (0.20—0.93)** | **0.03** |
| Baseline Symptomatic (ref = No) | 1.52 (0.50—4.58) | 0.46 | 2.50 (0.70—8.87) | 0.16 |
| Stricture Type on Endoscopy (ref = Fibrotic) | 1.38 (0.63—3.02) | 0.43 | 1.37 (0.53-3.58) | 0.52 |
| Ability to Pass Scope Before Dilatation (ref = No) | 0.60 (0.14—2.62) | 0.49 | 0.24 (0.03—1.94) | 0.18 |
| Technical Success x Stricture Type (ref = No, De-novo) | 0.83 (0.17—4.04) | 0.82 | 11.3 (0.94—135.0) | 0.06 |

**Supplementary Table 6**: Risk of CD-related surgery and hospitalization in patients with Crohn’s disease including an interaction between Technical success and Symptomatic vs Asymptomatic Strictures.

| **Characteristic** | **CD-related Surgery** |  |
| --- | --- | --- |
|  | **HR (95% CI)** | **p-value** |
| Technical Success (ref = No) | 0.21 (0.02—2.43) | 0.21 |
| Max Dilation (mm) | 1.05 (0.81—1.36) | 0.70 |
| Age | 0.99 (0.96-1.01) | 0.28 |
| Sex (ref = M) | 1.50 (0.73—3.08) | 0.27 |
| Stricture Type (ref = Denovo) | 0.62 (0.28—1.39) | 0.25 |
| Smoke (ref = Never) |  |  |
| Current | 1.62 (0.68—3.82) | 0.28 |
| Former | 1.94 (0.73—5.16) | 0.19 |
| Multiple Strictures (ref = No) | **2.49 (1.15—5.42)** | **0.02** |
| Baseline Advanced Therapy (ref = No) | 1.06 (0.49—2.27) | 0.88 |
| Baseline Symptomatic (ref = No) | 1.07 (0.12—9.35) | 0.95 |
| Stricture Type on Endoscopy (ref = Fibrotic) | 1.37 (0.62—3.01) | 0.44 |
| Ability to Pass Scope Before Dilatation (ref = No) | 0.59 (0.13—2.56) | 0.48 |
| Technical Success x Stricture Type (ref = No, De-novo) | 1.58 (0.13—18.6) | 0.72 |

**Supplementary Table 7:** Relative Risk for repeated dilations within 6 months.

| **Characteristic** | **Repeated Dilation in 6 months** |  |
| --- | --- | --- |
|  | **RR (95% CI)** | **p-value** |
| Technical Success (ref = No) | 1.04 (0.89—1.21) | 0.64 |
| Max Dilation (mm) | 1.00 (0.96—1.04) | 0.97 |
| Age | 1.00 (1.00—1.00) | 0.68 |
| Sex (ref = M) | 1.00 (0.91—1.09) | 0.96 |
| Stricture Type (ref = Denovo) | 1.03 (0.93—1.14) | 0.59 |
| Smoke (ref = Never) |  |  |
| Current | 1.05 (0.93—1.18) | 0.44 |
| Former | 1.01 (0.89—1.14) | 0.92 |
| Multiple Strictures (ref = No) | **0.89 (0.81—0.98)** | **0.02** |
| Baseline Advanced Therapy (ref = No) | 1.07 (0.98—1.18) | 0.14 |
| Baseline Symptomatic (ref = No) | **1.12 (1.02—1.23)** | **0.02** |
| Stricture Type on Endoscopy (ref = Fibrotic) | 1.02 (0.93—1.12) | 0.73 |
| Ability to Pass Scope Before Dilatation (ref = No) | 0.93 (0.80—1.08) | 0.32 |
